# Workload implications for clinic workflow with implementation of three-dimensional printed customized bolus for radiation therapy: A pilot study

**DOI:** 10.1371/journal.pone.0204944

**Published:** 2018-10-01

**Authors:** Eric Ehler, David Sterling, Kathryn Dusenbery, Jessica Lawrence

**Affiliations:** 1 Department of Radiation Oncology, Medical School, University of Minnesota, Minneapolis Minnesota, United States of America; 2 Department of Veterinary Clinical Sciences, College of Veterinary Medicine, University of Minnesota, St Paul, Minnesota, United States of America; RMIT University, AUSTRALIA

## Abstract

Bolus is commonly used in radiation therapy to improve radiation dose distribution to the target volume, but commercially available products do not always conform well to the patient surface. Tumor control may be compromised, particularly for superficial tumors, if bolus does not conform well and air gaps exist between the patient surface and the bolus. Three-dimensional (3D) printing technology allows the creation of highly detailed, variable shaped objects, making it an attractive and affordable option for customized, patient-specific bolus creation. The use of 3D printing in the clinical setting remains limited. Therefore, the objective of this study was to assess the implications on time and clinical fit using a workflow for 3D printing of customized bolus in companion animals with spontaneous tumors treated with radiation therapy. The primary aim of this study was to evaluate the time required to create a clinical 3D printed bolus. The secondary aims were to evaluate the clinical fit of the bolus and to verify the skin surface dose. Time to segmentation and 3D printing were documented, while the clinical fit of the bolus was assessed in comparison to the bolus created in the treatment planner. The mean and median time from segmentation to generation of 3D printed boluses was 6.15 h and 5.25 h, respectively. The 3D printed bolus was significantly less deviated from the planned bolus compared to the conventional bolus (p = 0.0078) with measured dose under the bolus within 5% agreement of expected dose in 88% of the measurements. Clinically acceptable 3D printed customized bolus was successfully created for treatment within one working day. The most significant impact on time is the 3D printing itself, which therefore has minimal implications on personnel and staffing. Quality assurance steps are recommended when implementing a 3D printing workflow to the radiotherapy clinic.

## Introduction

Bolus is commonly used in radiation therapy to improve radiation dose distribution to the target volume in order to increase the likelihood of tumor control.[1] Commercially available bolus is available which consists of elastic-like flexible sheets comprised of a uniform thickness (SuperFlab, Radiation Products Design, Inc., Albertville, MN or Skinless Bolus, CIVCO Medical Solutions, Kalona, IA) however these sheets do not conform well to complex anatomical contours.[1–3] Alternative materials such as thermoplastic material, wet gauze and Play-Doh (Hasbro, Pawtucket, RI) may be used to manually create bolus, however they require time for construction, may not precisely match the treatment plan, and often lack conformity to the patient surface.[1, 3–6] Bolus is used specifically to help create radiation isodose curves that optimally cover the radiation target; if it does not conform well to the patient’s surface and results in air gaps, tumor control may be compromised, particularly for superficial target volumes.[1, 7–9] Once bolus is determined to be necessary for the treatment plan, it is often clinic staff or therapists who prepare the bolus for use based on instructions from the radiation oncologist. This introduces a level of uncertainty in the creation and use of the bolus in clinical practice.[1]

Three-dimensional (3D) printing technology has evolved rapidly and allows the creation of highly detailed, variable shaped objects. There is tremendous interest in utilizing 3D printing for radiotherapy, in part due to improved access to affordable printers, the development of novel printing materials, and early achievements in surgical oncology and medical imaging.[2, 3, 10–14] Research in 3D printing for bolus has expanded tremendously with several reports describing the nonclinical evaluation of bolus.[15–20] While there is tremendous potential for the routine use of 3D printing in the daily treatment of radiotherapy patients, its use in the clinical setting remains limited.[3, 14, 19, 21, 22] The purpose of this study was to assess the implications on time and clinical fit while establishing a workflow for 3D printing of customized bolus in companion animals with spontaneous tumors that were treated with radiation therapy. The variation in the shape and size of dogs and cats provides a unique and relevant model in which to assess the potential applications of 3D printing in radiotherapy. While size, shape and conformation differ between companion animals and humans, the similarities in therapeutic goals, treatment planning, and external beam delivery methods create an ideal clinical setting in which to assess the implications of the 3D printing workflow. The primary aim was to evaluate the time required to create a clinical 3D printed bolus while the secondary aims were to evaluate the clinical fit of the bolus and to verify the skin surface dose.

## Methods

### Clinical cases

Canine and feline veterinary patients with spontaneous occurring, histologically confirmed cancer that presented to the Oncology Service between September 2016 and December 2017 were eligible to be enrolled in a prospective clinical study to assess the efficacy of 3D printed radiotherapy bolus. The study was approved by the University of Minnesota Institutional Animal Care and Use Committee (IACUC, Protocol No. 1608-34034A), and written client consent was obtained for all companion animals enrolled.

#### Computed tomography (CT) image acquisition and radiation planning

CT images were acquired on a helical 64-slice CT scanner (Toshiba Aquilion 64 CFX, Toshiba Medical Systems, Tustin, CA). CT scans were performed with animal patients in radiation therapy planning position, which was determined by the radiation oncologist and dependent on the tumor location and geared towards maximizing the reproducibility of repeated setup. All examinations consisted of reconstructed 2- or 3-mm-thick transverse images. Intravenous contrast medium (770 mg or I/kg; Optiray 350 [Ioversol], Mallinckrodt Inc, Hazelwood, MO) was administered to all animals following acquisition of pre-contrast data. Non contrast- and contrast-enhanced CT studies of the tumor region were retrieved from a PACS workstation in DICOM file format and sent to a computer utilizing commercially available contouring software (MIM Maestro 6.12, MIM Software Inc., Cleveland OH). Prior to radiation therapy, contours were manually drawn by the radiation oncologist that outlined regions of interest (ROI), tumor / target volumes and any nearby critical normal structures (segmentation). For tumors in the skin/subcutaneous tissue, bolus was created and retracted from the skin edge by 1 mm. Image sets with RT structure sets were subsequently sent from MIM to the treatment planning station (Pinnacle^3^, Philips Oncology Systems, Fitchburg, WI).

### 3D printing of the radiation modifier

During target segmentation, a bolus structure was created and a density override was applied to the bolus structure in the dose calculation. The bolus structure was designed to allow a 1 mm margin from the skin to bolus to compensate for fur that is not apparent on the CT scan. The bolus structure was exported from the segmentation software as a DICOM-RT file and converted to a .stl (stereolithography) file. The .stl file was loaded in 3D modeling software (Netfabb, Autodesk Inc, San Rafael CA). The 3D model was inspected for proper conversion from DICOM-RT format to .stl and if necessary, modification to the model was performed. All models were smoothed for 5 iterations with “prevent model shrinking” set to off.

For all cases, the plastic used was Polyethylene Terephthalate–Glycol-Modified, PETG, (eSun, Shenzhen Esun Industrial Co. Ltd., Shenzhen, China). Printing was performed on a Fused Deposition Modelling type 3D printer (Taz 6, Aleph Objects, Inc., Loveland CO). Initially, a nozzle with a 0.5 mm extrusion diameter was used (Case 1 through Case 11) and later, for Case 12 through Case 14, the nozzle was upgraded to 1.2 mm extrusion diameter (MOARstruder, Aleph Objects, Inc., Loveland CO). The larger extrusion diameter allowed for reduced print times due to two factors: faster extrusion through the larger diameter opening and larger layer heights resulting in fewer printing layers. The layer heights were 0.3 mm for the 0.5 mm nozzle and 0.6 mm for the 1.2 mm nozzle. The bolus for Case 11 was originally printed with the standard nozzle and after the upgrade the bolus was reprinted with the upgraded nozzle in order to compare print times for the same 3D model. All printing parameters were kept constant when possible for the 0.5 mm and 1.2 mm nozzle print settings. To that end the standard print settings supplied with the printer for nGen (a proprietary PET-G) were used with minor modifications for both extruders.

#### Time to 3D printed bolus generation

The time required to generate each 3D printed bolus was recorded for all cases. The process was divided into the following subroutines: bolus segmentation, conversion from DICOM-RT structure to 3D model (including model processing and smoothing), and 3D print time. Time reporting was limited to 0.25 hour (h) increments except for 3D print time, which was recorded by the printer precisely to the minute.

### Spatial accuracy of bolus placement for treatment

**Fig 1:**
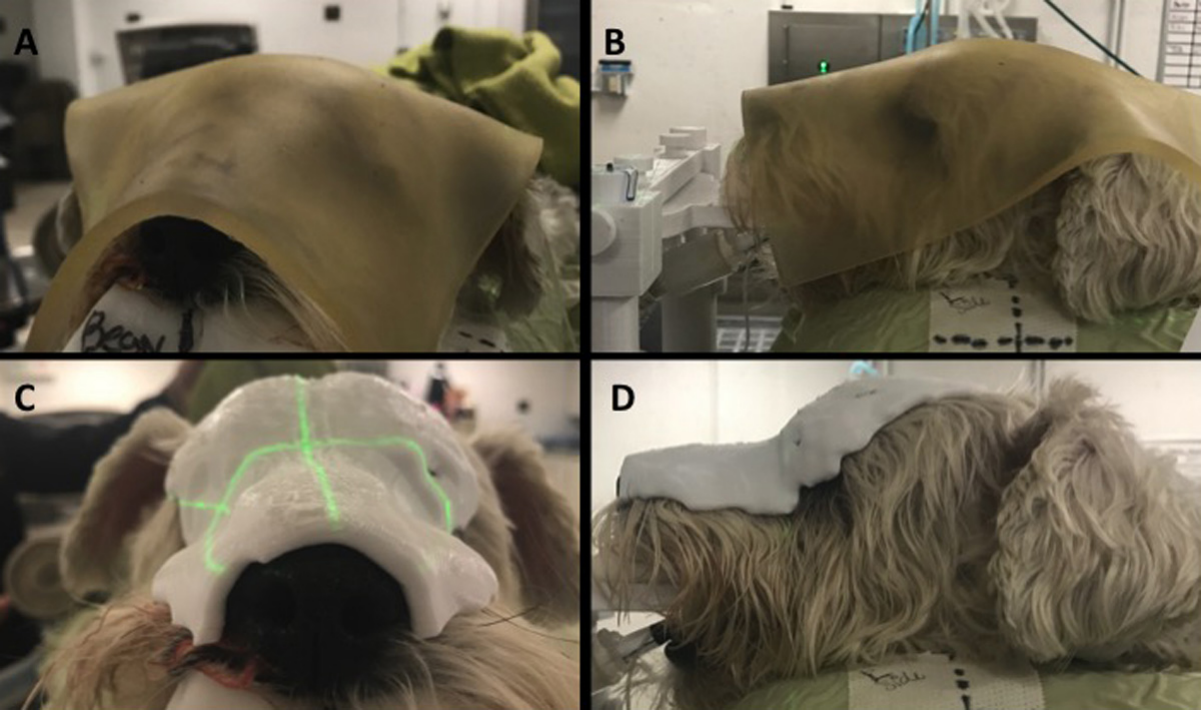
Photographs of the commercially available skinless bolus (**A** and **B**) in place prior to treatment compared to the customized 3D bolus in the dame dog (**C** and **D**).

Immediately prior to each radiation fraction, in order to verify proper patient positioning, kilovolatge (kV) CT (cone beam CT; CBCT) images on board the linear accelerator (Varian iX, Varian Medical Systems, Palo Alto CA) were obtained prior to each prescribed fraction in order to verify that patient setup and positioning match to the treatment-planning position. Conventional bolus (wet gauze or skinless bolus [CIVCO Medical Solutions]) was planned for all cases and used on the first treatment unless the 3D printed bolus was deemed superior by the clinician. The radiation modifier was in place at the time of CBCT, permitting an assessment of conformation, or clinical fit, to the patient’s external contour (Fig 1). At least one CBCT with the 3D printed bolus was planned for all clinical patients during the time of the study. The pretreatment CBCT was exported and the bolus was segmented in the CBCT scan. The CBCT was fused to the treatment planning CT using internal anatomy nearest the target volume. The segmented 3D printed bolus was compared to the bolus structure from the planning CT using mean surface deviation. Contouring on CBCT images and the mean surface deviation was performed on Velocity (Velocity v3.1.0, Varian Medical Systems, Inc., Palo Alto, CA) segmentation software. The conventional bolus was also segmented on CBCT and compared. If the conventional bolus was much larger than the original bolus structure from the planning CT (i.e. the sheet of bolus was not trimmed down to a patient-specific size), the excess bolus was not included in the CBCT conventional bolus structure to avoid inappropriately penalizing the conformality score of the conventional bolus. In addition, the density of the 3D printed bolus was determined by first calculating the weight of the bolus using a calibrated scale and subsequently determining the ratio of the weight to volume of the 3D model.

#### In-vivo dosimetry

To verify dose build-up under the bolus, in-vivo dosimetry measurements were performed between the bolus and skin using either radiochromic film (EBT3, Ashland Advanced Materials, Bridgewater, NJ) or Optically Stimulated Luminescence Detectors (OSLD). The radiation oncologist provided the expected dose while the physicist determined the measured dose. Expected measurements were obtained by registering the film location on the cone beam CT for setup with the treatment planning CT illustrating dose distribution. Films were calibrated using triple channel dosimetry and fit to a rational function.[23] The calibration dose ranged from 0 to 800 cGy. Film orientation was denoted on the films to ensure consistency in film orientation with the calibration film orientations to reduce uncertainties. Films were scanned on a flatbed 48-bit scanner (Epson Expression 11000XL, Seiko Epson Corp., Suwa Japan) at a resolution of 72 DPI. Film dose values are reported as the mean dose to a defined region of interest on the film. OSLD were calibrated and read out to manufacturer specification (Landauer Microstar, Landauer Inc., Glenwood IL). Films were cut but the minimum size was limited to 1 cm by 1 cm to prevent edge effects, such as delamination, from influencing measured dose.

#### Data analysis

Time to print the bolus versus the volume of bolus was correlated using Spearman’s coefficient (Graphpad Prism v.7, La Jolla CA). Mean surface deviation differences between the 3D bolus and conventional bolus and differences between measured dose and expected dose were determined by Wilcoxon rank test (GraphPad Prism). Statistical significance was set at p < 0.05.

## Results

### Clinical use of 3D bolus

#### Clinical case characteristics

Fourteen companion animals were enrolled in the study. Clinical details are included in Table 1. Twelve dogs and two cats were included over 14.5 months, with various breeds, animal size and tumor location represented. One case (Case 3) experienced a partial response during definitive-intent radiation therapy, resulting in the formation of an air gap between the bolus and the tumor mass determined on CBCT. Using a daily CBCT image, an insert was designed and printed as a compensator in order to fill the air gap resulting from tumor regression, which avoided the need for a repeat CT scan for re-planning. The initial bolus for this dog is hereafter referred to as Case 3a while the initial bolus in combination with the insert (compensator) is referred to Case 3b. All patients tolerated the bolus well and placement by trained veterinary technicians was simple and easily assessed on the CBCT. All patients were treated with 3D conformal radiation therapy with 6MV photons prescribed to isocenter within the planning target volume (PTV).

**Table 1:** Clinical characteristics of companion animals treated with 3D printed bolus during radiation therapy.

Animal	Breed	Weight (kg)	Tumor Type	Tumor Location	Fractional Dose to Isocenter (cGy)	Total Dose (cGy)
1	Toy Poodle	4.8	Carcinoma	Nasal	600	2400
2	Toy Poodle	2.5	Carcinoma	Perianal	250	5000
3	Jack Russell terrier	5.9	Sarcoma (peripheral nerve sheath tumor)	Sacral	270	5400
4	Cocker spaniel cross	11.6	Melanoma	Maxillary	600	2400
5	Miniature Schanuzer	10.1	Carcinoma	Nasal	600	2400
6	Siberian Husky	17.1	Squamous cell carcinoma	Maxillary	250	5000
7	French bulldog	12.2	Squamous cell carcinoma	Maxillary	250	5000
8	French bulldog	9.6	Sarcoma (peripheral nerve sheath tumor)	Cervical spinal cord	250	5000
9	Golden Retriever	29.1	Mast cell tumor	Ventral cervical skin	250	5000
10	Rhodesian Ridgeback	45.5	Carcinoma	Nasal	270	4860
11	Mixed breed dog	25.1	Osteosarcoma	Maxillary	600	2400
12	Domestic short hair cat	7.6	Adenocarcinoma	Nasal	270	4860
13	Golden Retriever	31.5	Fibrosarcoma	Maxillary	280	5600
14	Domestic short hair cat	5.0	Adenocarcinoma	Nasal	270	4860

#### Bolus generation time

**Fig 2:**
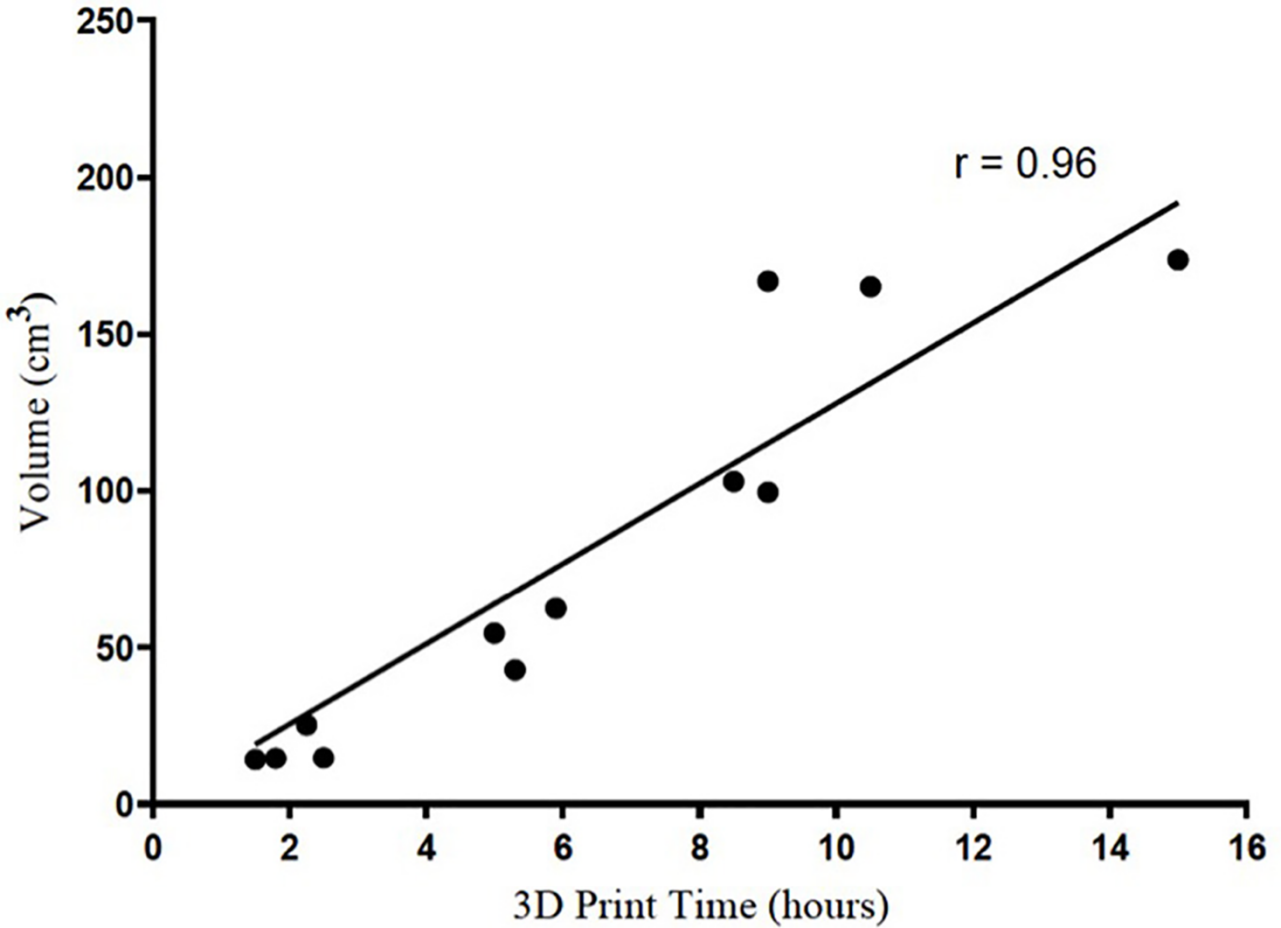
Correlation of time required to 3D print bolus to bolus volume. The hours required to generate 3D printed bolus was significantly correlated to the size of the bolus.

The time required to print each 3D bolus is shown in Table 2. The mean and median time for bolus segmentation were both 1 h, while the mean and median time to convert the segmented bolus to a 3D model was 0.7 h and 0.5 h, respectively. The mean and median time to print all customized boluses was 5.25 h and 4.25 h, respectively (range 0.6 h– 10.5 h). Taking the overall time in account, the mean and median time from segmentation to generation of boluses was 6.15 h and 5.25 h, respectively. Finally, when only the initial small nozzle was considered (Case 1 through Case 11), the mean and median time to bolus creation from segmentation to printing was 5.6 h and 6.35 h, respectively. A bolus construction rate of 60 cm^3^/h was achieved with the 1.2 mm diameter nozzle as opposed to a maximum rate of 18.5 cm^3^/h for the 0.5 mm nozzle. The bolus for one case (Case 11) was printed with each nozzle for comparison; the print time was reduced from 10.5 h to 3.0 h with use of the upgraded nozzle. Time to print the bolus was significantly correlated to the volume of bolus created (Fig 2; p < 0.0001). The median and mean print density for all boluses created was 1.16 g/cm^3^ and 1.15 g/cm^3^, respectively, with a range from 1.11 g/cm^3^ to 1.20 g/cm^3^. When separated to nozzle size, bolus printed with the 0.5 mm nozzle had a mean density of 1.14 g/cm^3^ (n = 12) while bolus printed with the 1.2 mm nozzle had a mean density of 1.19 g/cm^3^ (n = 4).

**Table 2:** Characteristics of 3D bolus generation.

Case	Bolus Segmentation (hours)	Conversion to 3D Model (hours)	3D Print Time (hours)	Volume (cm^3^)	Printer Rate (cm^3^/hour)	Density (g cm^-3^)
1	2.5	1.0	1.8	14.7	8.2	1.12
2	1.0	0.5	1.5	14.3	9.5	1.11
3a	0.5	0.5	8.5	103.1	12.1	1.16
3b	0.5	0.5	2.5	14.81	5.9	1.20
4	2.0	1.0	2.25	25.4	11.3	1.15
5	1.0	1.0	5.0	54.7	10.9	1.11
6	1.0	1.0	9.0	166.9	18.5	1.12
7	2.0	1.0	5.3	42.9	8.1	1.13
8	2.0	1.0	5.9	62.6	10.6	1.14
9	1.0	1.0	15	173.7	11.6	1.17
10	0.5	0.5	9.0	99.64	11.1	1.16
11	0.5	0.5	10.5	165.2	15.7	1.13
11	0.5	0.5	3.0	165.2	55.1	1.17
12	0.5	0.5	0.75	20	26.7	1.19
13	0.5	0.5	3.5	222	63.4	1.20
14	0.5	0.5	0.6	19.8	33.0	1.19

Boluses printed with an upgraded 1.2 mm extrusion diameter nozzle are shaded grey while remaining boluses were printed with a 0.5 mm extrusion diameter nozzle.

### Positional accuracy and dose buildup of 3D printed bolus

**Fig 3:**
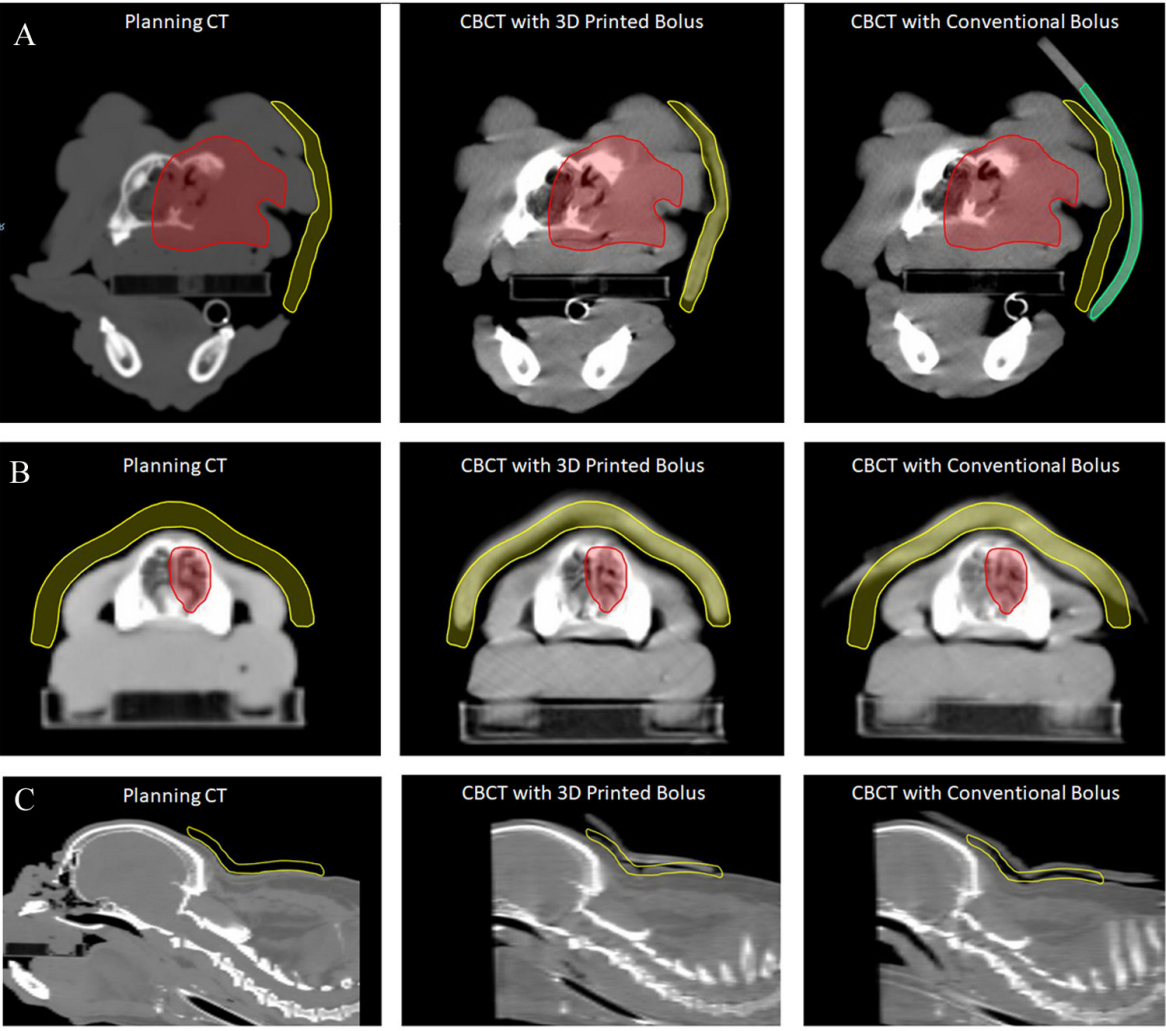
Spatial accuracy of 3D bolus placement compared to the planned bolus. The planned bolus is in yellow while the PTV is in red where visible. Images demonstrate the planned bolus (left), 3D generated bolus on the CBCT (middle) and conventional bolus on the CBCT (right). (A) Axial images demonstrate bolus for Case 7, which was the largest mean surface deviation for the conventional bolus (far right) compared to the planned bolus. The conventional bolus is outlined in green here to demonstrate its location external to the planned bolus. (B) Bolus for Case 14, which demonstrated the smallest mean surface deviation for the conventional bolus. (C) Bolus for Case 9, which generated a poor fit with both 3D printed bolus and conventional bolus due to a large number of mobile skin folds.

The positional accuracy of each 3D printed bolus was assessed via evaluation of the planned bolus from the planning CT and the actual position during CBCT for daily radiation setup verification (Fig 3).

**Fig 4:**
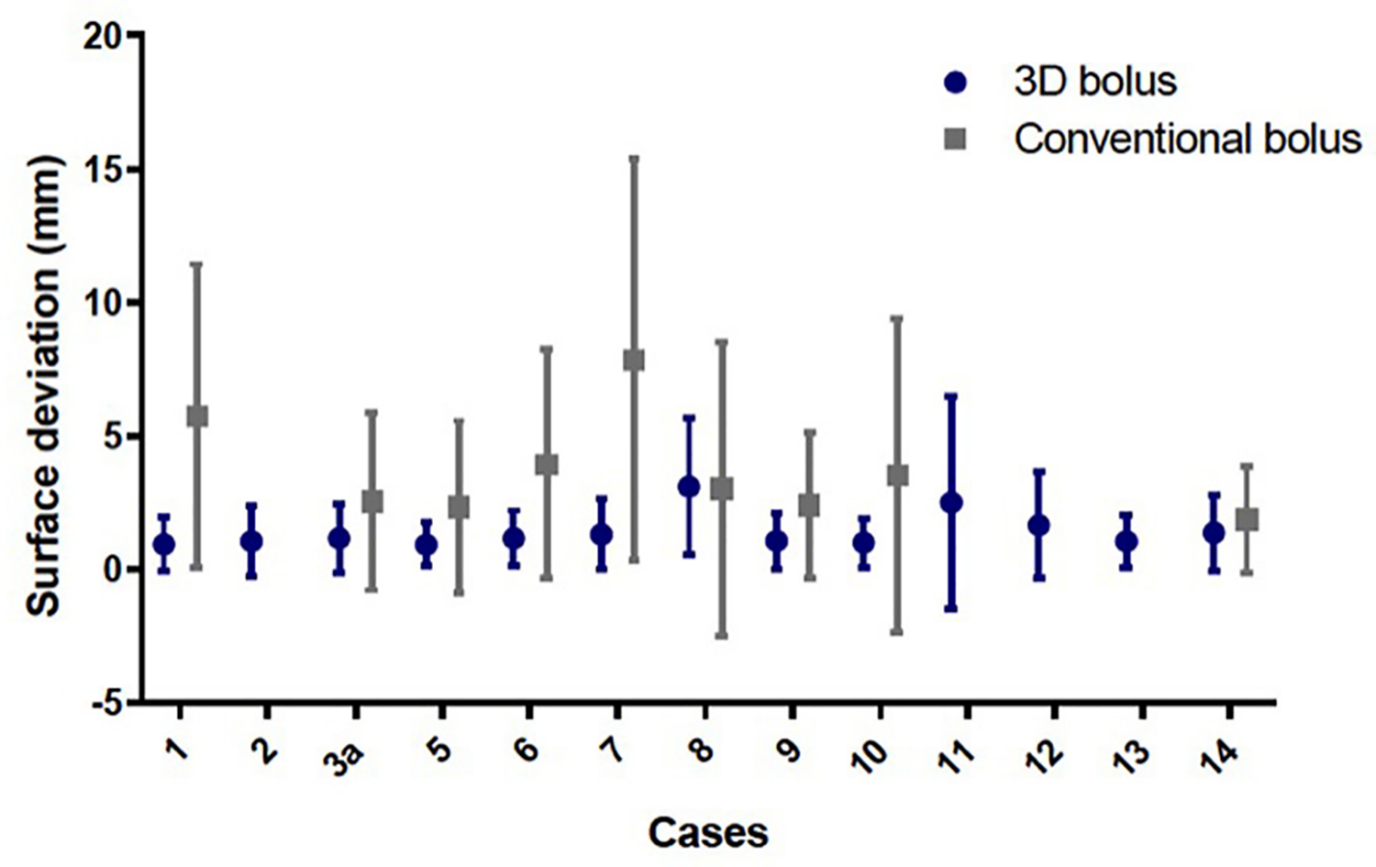
Mean surface deviation in the treatment bolus compared to the planning bolus. The mean surface deviation in the 3D bolus or the conventional bolus is presented for each case as the mean deviation in mm with the standard deviation (SD).

The mean surface deviation of actual 3D printed bolus position versus the planning system bolus position is summarized in Table 3. It was not possible to observe the positional accuracy of bolus for one patient due to the fact that it was outside the cranial-caudal limits of the CBCT. In that case, the bolus was used under the cervical region on a dog to create bolus for a lymph node chain that was too far from isocenter, resulting in the bolus being outside of the CBCT. For comparison, the mean surface deviation of the conventional bolus for nine patients is shown in Table 3. For 4 cases, an acceptable conventional bolus using wet gauze or bolus was not deemed suitable for the patient and thus only the 3D printed bolus was used. Only 1 case (Case 8) was compared with wet gauze while the remaining cases were treated with commercially available skinless bolus. While an attempt was made to treat all cases using skinless bolus to mirror common practice in human radiation oncology, Case 8 was a brachycephalic dog with several skin folds over the treatment region. Wet gauze provided better conformal ability over the skin folds while the skinless bolus had poor contact. The 3D bolus was significantly less deviated from the planned bolus compared to the conventional bolus (p = 0.0078); the mean surface deviation was 1.40 mm ± 1.51 mm for the 3D printed bolus (n = 13) versus 4.15 mm ± 5.09 mm for conventional bolus (n = 9) based upon measurements obtained from CBCT scans (Fig 4). The conventional bolus only demonstrated a lower mean surface deviation over the 3D printed bolus in one case (Case 8). Notably, of the 9 cases with both CBCT of the 3D printed bolus and conventional bolus for side-by-side evaluation, there was a difference in the mean surface deviation of at least 1 mm in 7 cases.

**Table 3:** Positional accuracy of the 3D printed bolus compared to the planning system bolus.

Case	3D Printed Bolus	Conventional Bolus
	Mean Surface Deviation (± SD) [mm]	Mean Surface Deviation (± SD) [mm]
1	0.93 ± 1.00	5.75 ± 5.68
2	1.05 ± 1.34	NA
3a	1.15 ± 1.30	2.53 ± 3.31
5	0.92 ± 0.81	2.34 ± 3.22
6	1.16 ± 1.03	3.93 ± 4.29
7	1.30 ± 1.30	7.84 ± 7.52
8	3.09 ± 2.58	2.99 ± 5.51
9	1.06 ± 1.04	2.39 ± 2.76
10	0.99 ± 0.92	3.51 ± 5.88
11	2.50 ± 4.00	NA
12	1.65 ± 1.99	NA
13	1.05 ± 0.96	NA
14	1.36 ± 1.40	1.86 ± 1.97

SD, standard deviation; NA, not available for measurement.

Dosimetry measurements at the skin/hair surface directly under the 3D printed bolus are shown in Table 4. Measurements were not significantly different (p = 0.71) and agreement within ± 5% was found for 14 of the 16 measurements recorded. The mean percentage difference from the expected dose was 0.2 ± 3.8%; the mean absolute difference was 3.2 ± 1.9%. Seven of the 16 measurements were greater than 1% below the expected dose, 7 were greater than 1% above the expected dose, and 2 measurements were within 1%.

**Table 4:** In vivo dosimetry measurements.

Case	Measured Dose	Expected Dose	% Difference
1	587	600	-2.2
2	264	270	-2.2
3a^a^	287	270	6.3
3b	260	270	-3.7
4	572	600	-4.7
5	507	490	3.5
6	200	200	0
7	215	200	7.5
8^a^	255	250	2.0
10	240	232	3.4
	241	238	1.3
11	538	560	-3.9
13	278	270	3.0
	273	283	-3.5
14	239	250	-4.4
	234	233	0.4

^a^denotes measurement with optically stimulated luminescence detector.

## Discussion

This is the first clinical study to document the clinical workload implications of 3D printing bolus material for use with routine radiation therapy cases. For all 14 cases included in the study, regardless of conformation or tumor location, 3D generated bolus was successfully generated and used for treatment within a 12 h window of time (Table 2). The greatest time factor in the workflow was the 3D printing, which does not involve direct human involvement for the vast majority of the time. Typically during the 3D printing process, the printer was observed while it performed pre-printing checks and printed the first layer to ensure proper operation of the printer. None of the print jobs failed during printing. In terms of human time required, the segmentation of the bolus took the greatest time. This time was spent between the clinician (JL) and the physicists (EE and DS). The clinician contoured bolus where it was needed for radiation planning to improve dose distribution; afterward the physicists would evaluate the bolus segmentation and modify it if necessary based on 3D printing considerations. Notably, the time required for bolus segmentation decreased as the individuals involved in the process gained experience, despite that this pilot study occurred over the course of only 14.5 months. The time requirement for this process was reduced to 0.5 hours for the last 5 cases. Likewise, the conversion of the bolus as a segmentation in a contouring software system to a 3D model in .stl format required less time with experience. For all cases, the time was 1.0 hours or less to perform this step and for cases that required greater than 0.5 hours, it was due to human error (i.e. an unintended hole in the bolus due to contouring on axial slices) that required revision of the bolus segmentation.

As expected, the 3D printed bolus resulted in better mean surface deviation of the actual bolus location when referenced to the planned bolus compared to the conventional bolus. Fig 3 demonstrates the extremes of the cases included, with the greatest and least mean surface deviation for conventional bolus. One reason for the large deviation in Case 7 is that the bolus was intended to conform to a highly irregular surface, specifically the maxilla and muzzle of a French bulldog. These brachycephalic breeds are characterized by short nasal cavities and abundant moveable skin folds that are challenging to recreate, and therefore to consistently fit a conventional bolus to their skin surface for repeated treatments. Moreover, the orientation of the patient and the desired location of the bolus did not allow for gravity to help keep the bolus in place spontaneously. The 3D printed bolus in this case performed clinically superiorly to the conventional bolus in part because it could help recreate skin folds and increase surface contact. While normal human anatomy may be more predictable and regular, superficial tumors may distort the skin surface in humans, thus this case illustrates a clear clinical scenario where 3D printed bolus improves treatment delivery. This may be particularly relevant when considering the need for posterior bolus in humans, as conventional bolus may fall during treatment and therefore not conform to the patient as desired. For bolus needed on lateral aspects of patients, bolus can help to recreate body curves and be secured in place with tape or anchored against an immobilization mattress. It has been demonstrated that air gaps of 10 mm can result in a 10% reduction in the surface dose for photons, but this may be less predictable for small fields treated with modulated fields.[1, 24, 25] Thus, 3D printed bolus may contribute dramatically to controlling tumors adjacent to the region of dose buildup.

Importantly, the upgraded, larger extruder resulted in equal conformity for Cases 12–14, representing that once a workflow is established, it can be fluid as technology allows for modifications. Qualitative comparison of the bolus for Case 11, printed with both extrusion diameters, showed little difference in the spatial qualities of the bolus but a much faster time to production. Considering the typical resolution of a planning CT is on the order of mm, a sub-mm change in nozzle diameter, while resulting in loss of printer resolution, does not seem to clinically affect the outcome of the bolus. Additionally, greater uniformity in bolus density with the larger extruder nozzle was noted (Table 2), which may affect quality control measures needed to assess each bolus for human use. It is clear that the greatest benefit of the larger extruder is the relevant impact on bolus generation. 3D customized, patient-specific bolus could be generated with just a one-day turnaround time, thus could be used even for urgent cases. For example, Case 13 had a 222 cubic centimeter volume bolus and the total time to create the bolus was 4.5 hours (3.5 hours to print). Compared to the smaller nozzle extruder, which required 15 hours to print a 174 cubic centimeter bolus, the 1.2 mm nozzle extruder greatly reduces the overall time to generate a bolus. In human radiation oncology, a typical bolus will likely be of equal or larger size and volume than the largest boluses generated for the majority of cases in this study. A common bolus used for human treatments is 40 cm by 40 cm by 0.5 cm; at the build rates observed with the 1.2 mm extrusion nozzle, an equivalent size 3D printed bolus would be constructed in approximately 14 hours.

Additional reduction in 3D print time may also be possible. A third bolus was generated for Case 11 with the 1.2 mm nozzle and a 1.2 mm layer height (rather than 0.6 mm) and the print time was further reduced to 1.7 hours. Compared to the other two boluses, no appreciable difference was seen in the density of the bolus (1.15 g cm^-3^) or CT in comparison of the boluses (mean surface deviation of 0.56 ± 0.36 mm); visually it was apparent the bolus was printed with a greater layer thickness. However, this additional bolus was not evaluated on the clinical cases presented here, but further investigation of 3D print time reduction is ongoing.

The printer settings used for bolus generation were based on the standard printer settings for nGen in the vendor supplied slicing software. The purpose of this was to assess whether staff with limited experience with 3D printing would be able to generate similar results. Modifications were designed to limit the use of support structures during 3D printing. Importantly, based on our study, the use of the generalized printer settings would not affect the outcome of the bolus conformity, suggesting minimal experience does not hinder adoption of 3D printing technology in most clinical settings. The financial implications for generating 3D printed bolus are modest following purchase of a printer; for example, the cost for 1 kg of the 3D printing material used in this study is $30 USD, which is sufficient to generate one 750 cm^3^ bolus (just under the volume of a 40 cm by 40 cm by 0.5 cm conventional bolus).

The printed densities ranged from 1.11 to 1.20 g cm^-3^, a variation under 10%. The principle reason for this variability was the creation of small air voids within the bolus. As the plastic was printed, small air gaps can develop, even when 100% infill is specified at the outset. This can partially be compensated for with printer settings and choice of slicing software. However, special care should be taken when using 3D printed bolus in both planning and with clinical use. For example, if the bolus was assumed to have a 1.0 g cm^-3^ density in the treatment plan and the actual printed density of the bolus was 1.2 g cm^-3^, a clinically relevant dose alteration could occur depending on the radiation modality and other planning parameters. Therefore, a clinical quality assurance program is required if 3D printed bolus is routinely used to ensure the 3D bolus performs as expected.

There were no significant differences between the expected and measured doses in this study, although it is important to recognize that clinical significance may not mirror statistical significance. Although toxicity was not an endpoint in this pilot study, unexpected toxicity did not develop in any case and all acute toxicities were predominantly resolved within 2–3 weeks of completion of treatment. A clear relationship was not identified between the difference between the measured dose difference and the 3D bolus conformity. For example, one case with the greatest mean surface deviation for the 3D printed bolus (Case 8) had better in vivo dosimetry agreement than a case with a lower mean surface deviation (Case 7; Fig 2A). This may in part be a reflection of the location of the in vivo dosimeters being on the skin surface rather than within the target volume, as they were highly prone to setup variability. One reason for the use of film was that it could be observed on even a small film if there was a high dose gradient on the dosimeter. Such information could not easily be determined from OSLD measurements.

It is important to reflect on the number of available printing materials that may be purchased and combined in the generation of 3D printed materials. We elected to use PETG in particular because of its low economic impact, our familiarity with its use in our printer, the robustness of the plastic to resist damage if dropped, the ability to easily clean the bolus, and the ability to mark the plastic during treatment to improve repeatability of setup. None of the 3D printed boluses sustained damage during treatment; one bolus fell from the couch to the floor (approximate 3 foot fall) during a rapid anesthetic recovery but remained intact. It is feasible that a thin 3D printed bolus could undergo damage if dropped or stepped on, thus extra care may be needed for these patient-specific modifiers. Other materials may be more suitable under some circumstances–PETG is firm and unyielding and thus may be uncomfortable for some human patients. In veterinary radiation oncology, companion animals are anesthetized for treatment to ensure they are positioned properly and remain immobile during treatment. Comfort of the bolus is difficult to assess in veterinary patients; however, none of the dogs and cats treated had altered anesthetic parameters following placement of the bolus. Additional material may prove more suitable for some human patients and more work needs to be done to address the infill, dosimetry and toxicity implications of other materials intended for use in radiation oncology. The use of thermoplastic elastomer (TPE) filament is attractive as it is a flexible plastic that can create elastic 3D printed materials that can be stretched but are not permanently deformed. Thermoplastic polyurethane (TPU) is a firmer variant of TPE that has been of interest for radiation oncology and includes NinjaFlex (NinjaTek, Manheim, PA) and Cheetah (NinjaTek). It is important that additional research defines specific radiologic properties and ideal conditions for use so that these materials can be worked in to the workflow safely.[20, 26, 27] A recent study found substantial variances in density between several commercially available materials including PLA, TPU plastic (NinjaFlex and Cheetah) and acrylonitrile butadiene styrene (ABS).[20] This density variation could affect clinical dose uncertainty; thus, the authors of that study support optimization of the 3D printing workflow within each clinic to account for differences in the printing process, radiation treatment planning system and CT scanner.[20] Additionally, from our initial experience with TPU plastic, it is not as reliable as PETG as it has a higher rate of print failure and requires more time to print; currently we have not successfully printed with TPU plastic on the 1.2 mm nozzle.

Limitations to the study include the small number of clinical patients investigated, the lack of dose verification under the conventional bolus, the lack of various thickness 3D printed bolus assessed, and that the time required to make conventional bolus was not accurately measured for comparison. It was not possible to fully assess the impact of time required to suitably select and adjust the conventional bolus used in this study due to the existing workflow in the veterinary clinic. The veterinary clinic reuses commercially available skinless bolus provided by the Department of Radiation Oncology at the Medical School following human treatment. The clinic therefore has a large collection of variably sized skinless bolus available for use that fits most animal cases and is selected and placed on the patient in under 5 minutes. For Case 8, in which wet gauze was used, it required approximately 3.5 minutes for the veterinary technician to build and confirm 0.5 cm thickness once wet. Comparing times could be evaluated in future studies, particularly in clinical scenarios where the same type of material is consistently used and standard sheets are shaped to suit the individual patient, as is done in many radiation clinics for patient use. However, the primary purpose of this initial study was to evaluate the time required to generate a 3D printed bolus to assess the impact on clinical workflow, thus we believe this data to be highly informative for future studies.

## Conclusion

The use of customized 3D printed bolus was successfully implemented using a workflow that generated a clinically acceptable patient-specific bolus for treatment within one working day, particularly with printer modifications for faster printing. The greatest time factor is the 3D printing itself, which does not require direct human involvement and therefore has minimal implications on personnel and staffing. Because conformity and bolus density and function should be assessed, quality assurance steps are recommended when implementing a 3D printing workflow to the radiotherapy clinic.

## References

[1] Vyas V, Palmer L, Mudge R, Jiang R, Fleck A, Schaly B, et al On bolus for megavoltage photon and electron radiation therapy. Med Dosim. 2013;38(3):268–73. Epub 2013/04/16. 10.1016/j.meddos.2013.02.007 . 23582702

[2] Chiu T, Tan J, Brenner M, Gu X, Yang M, Westover K, et al Three-dimensional printer-aided casting of soft, custom silicone boluses (SCSBs) for head and neck radiation therapy. Pract Radiat Oncol. 2018;8(3):e167–e74. Epub 2018/02/18. 10.1016/j.prro.2017.11.001 . 29452869

[3] Zhao Y, Moran K, Yewondwossen M, Allan J, Clarke S, Rajaraman M, et al Clinical applications of 3-dimensional printing in radiation therapy. Med Dosim. 2017;42(2):150–5. Epub 2017/05/13. 10.1016/j.meddos.2017.03.001 . 28495033

[4] Benoit J, Pruitt AF, Thrall DE. Effect of wetness level on the suitability of wet gauze as a substitute for Superflab as a bolus material for use with 6 mv photons. Veterinary radiology & ultrasound: the official journal of the American College of Veterinary Radiology and the International Veterinary Radiology Association. 2009;50(5):555–9. Epub 2009/10/01. . 19788044 10.1111/j.1740-8261.2009.01573.x

[5] Nagata K, Lattimer JC, March JS. The electron beam attenuating properties of SuperFlab, Play-Doh, and wet gauze, compared to plastic water. Veterinary radiology & ultrasound: the official journal of the American College of Veterinary Radiology and the International Veterinary Radiology Association. 2012;53(1):96–100. Epub 2011/11/19. 10.1111/j.1740-8261.2011.01866.x . 22092982

[6] Yoon J, Xie Y, Zhang R. Evaluation of surface and shallow depth dose reductions using a Superflab bolus during conventional and advanced external beam radiotherapy. J Appl Clin Med Phys. 2018;19(2):137–43. Epub 2018/02/11. 10.1002/acm2.12269 ; PubMed Central PMCID: PMCPMC5849823. 29427312 PMC5849823

[7] Kong M, Holloway L. An investigation of central axis depth dose distribution perturbation due to an air gap between patient and bolus for electron beams. Australas Phys Eng Sci Med. 2007;30(2):111–9. Epub 2007/08/09. . 17682400 10.1007/BF03178415

[8] Sharma SC, Johnson MW. Surface dose perturbation due to air gap between patient and bolus for electron beams. Med Phys. 1993;20(2 Pt 1):377–8. Epub 1993/03/01. 10.1118/1.597079 . 8497226

[9] Behrens CF. Dose build-up behind air cavities for Co-60, 4, 6 and 8 MV. Measurements and Monte Carlo simulations. Phys Med Biol. 2006;51(22):5937–50. Epub 2006/10/28. 10.1088/0031-9155/51/22/015 . 17068375

[10] Mitsouras D, Liacouras P, Imanzadeh A, Giannopoulos AA, Cai T, Kumamaru KK, et al Medical 3D Printing for the Radiologist. Radiographics. 2015;35(7):1965–88. Epub 2015/11/13. 10.1148/rg.2015140320 ; PubMed Central PMCID: PMCPMC4671424. 26562233 PMC4671424

[11] Matsumoto JS, Morris JM, Foley TA, Williamson EE, Leng S, McGee KP, et al Three-dimensional Physical Modeling: Applications and Experience at Mayo Clinic. Radiographics. 2015;35(7):1989–2006. Epub 2015/11/13. 10.1148/rg.2015140260 . 26562234

[12] Giannopoulos AA, Steigner ML, George E, Barile M, Hunsaker AR, Rybicki FJ, et al Cardiothoracic Applications of 3-dimensional Printing. J Thorac Imaging. 2016;31(5):253–72. Epub 2016/05/06. 10.1097/RTI.0000000000000217 ; PubMed Central PMCID: PMCPMC4993676. 27149367 PMC4993676

[13] Morrison RJ, Hollister SJ, Niedner MF, Mahani MG, Park AH, Mehta DK, et al Mitigation of tracheobronchomalacia with 3D-printed personalized medical devices in pediatric patients. Sci Transl Med. 2015;7(285):285ra64 Epub 2015/05/01. 10.1126/scitranslmed.3010825 ; PubMed Central PMCID: PMCPMC4495899. 25925683 PMC4495899

[14] Canters RA, Lips IM, Wendling M, Kusters M, van Zeeland M, Gerritsen RM, et al Clinical implementation of 3D printing in the construction of patient specific bolus for electron beam radiotherapy for non-melanoma skin cancer. Radiotherapy and oncology: journal of the European Society for Therapeutic Radiology and Oncology. 2016;121(1):148–53. Epub 2016/10/27. 10.1016/j.radonc.2016.07.011 . 27475278

[15] Fujimoto K, Shiinoki T, Yuasa Y, Hanazawa H, Shibuya K. Efficacy of patient-specific bolus created using three-dimensional printing technique in photon radiotherapy. Phys Med. 2017;38:1–9. Epub 2017/06/15. 10.1016/j.ejmp.2017.04.023 . 28610688

[16] Kim SW, Shin HJ, Kay CS, Son SH. A customized bolus produced using a 3-dimensional printer for radiotherapy. PloS one. 2014;9(10):e110746 Epub 2014/10/23. 10.1371/journal.pone.0110746 ; PubMed Central PMCID: PMCPMC4206462. 25337700 PMC4206462

[17] Park JW, Oh SA, Yea JW, Kang MK. Fabrication of malleable three-dimensional-printed customized bolus using three-dimensional scanner. PloS one. 2017;12(5):e0177562 Epub 2017/05/12. 10.1371/journal.pone.0177562 ; PubMed Central PMCID: PMCPMC5426771. 28494012 PMC5426771

[18] Ricotti R, Ciardo D, Pansini F, Bazani A, Comi S, Spoto R, et al Dosimetric characterization of 3D printed bolus at different infill percentage for external photon beam radiotherapy. Phys Med. 2017;39:25–32. Epub 2017/07/18. 10.1016/j.ejmp.2017.06.004 . 28711185

[19] Su S, Moran K, Robar JL. Design and production of 3D printed bolus for electron radiation therapy. J Appl Clin Med Phys. 2014;15(4):4831 Epub 2014/09/11. 10.1120/jacmp.v15i4.4831 ; PubMed Central PMCID: PMCPMC5875499. 25207410 PMC5875499

[20] Craft DF, Kry SF, Balter P, Salehpour M, Woodward W, Howell RM. Material matters: Analysis of density uncertainty in 3D printing and its consequences for radiation oncology. Med Phys. 2018;45(4):1614–21. Epub 2018/03/02. 10.1002/mp.12839 . 29493803

[21] Lukowiak M, Jezierska K, Boehlke M, Wiecko M, Lukowiak A, Podraza W, et al Utilization of a 3D printer to fabricate boluses used for electron therapy of skin lesions of the eye canthi. J Appl Clin Med Phys. 2017;18(1):76–81. Epub 2017/03/16. 10.1002/acm2.12013 ; PubMed Central PMCID: PMCPMC5689892. 28291910 PMC5689892

[22] Park JW, Yea JW. Three-dimensional customized bolus for intensity-modulated radiotherapy in a patient with Kimura's disease involving the auricle. Cancer Radiother. 2016;20(3):205–9. Epub 2016/03/30. 10.1016/j.canrad.2015.11.003 . 27020714

[23] Lewis D, Chan M, Micke A, Yu X. SU-E-T-165: Protocol for Simplified Radiochromic Film Dosimetry. Med Phys. 2012;39(6Part12):3741 Epub 2012/06/01. 10.1118/1.4735223 . 28517799

[24] Butson MJ, Cheung T, Yu P, Metcalfe P. Effects on skin dose from unwanted air gaps under bolus in photon beam radiotherapy. Radiation Measurements. 2000;32(3):201–2014.

[25] Rustgi AK, Samuels A, Rustgi SN. Influence of air inhomogeneities in radiosurgical beams. Med Dosim. 1997;22(2):95–100. Epub 1997/07/01. . 9243461 10.1016/s0958-3947(97)00001-0

[26] Madamesila J, McGeachy P, Villarreal Barajas JE, Khan R. Characterizing 3D printing in the fabrication of variable density phantoms for quality assurance of radiotherapy. Phys Med. 2016;32(1):242–7. Epub 2015/10/29. 10.1016/j.ejmp.2015.09.013 . 26508016

[27] Dancewicz OL, Sylvander SR, Markwell TS, Crowe SB, Trapp JV. Radiological properties of 3D printed materials in kilovoltage and megavoltage photon beams. Phys Med. 2017;38:111–8. Epub 2017/06/15. 10.1016/j.ejmp.2017.05.051 . 28610691

